# Prenatal Malnutrition-Induced Epigenetic Dysregulation as a Risk Factor for Type 2 Diabetes

**DOI:** 10.1155/2019/3821409

**Published:** 2019-02-28

**Authors:** Alexander Vaiserman, Oleh Lushchak

**Affiliations:** ^1^Laboratory of Epigenetics, D.F. Chebotarev Institute of Gerontology, 67 Vyshgorodska, 04114 Kyiv, Ukraine; ^2^Department of Biochemistry and Biotechnology, Vasyl Stefanyk Precarpathian National University, 57 Shevchenko, 76018 Ivano-Frankivsk, Ukraine

## Abstract

Type 2 diabetes (T2D) is commonly regarded as a disease originating from lifestyle-related factors and typically occurring after the age of 40. There is, however, consistent experimental and epidemiological data evidencing that the risk for developing T2D may largely depend on conditions early in life. In particular, intrauterine growth restriction (IUGR) induced by poor or unbalanced nutrient intake can impair fetal growth and also cause fetal adipose tissue and pancreatic *β*-cell dysfunction. On account of these processes, persisting adaptive changes can occur in the glucose-insulin metabolism. These changes can include reduced ability for insulin secretion and insulin resistance, and they may result in an improved capacity to store fat, thereby predisposing to the development of T2D and obesity in adulthood. Accumulating research findings indicate that epigenetic regulation of gene expression plays a critical role in linking prenatal malnutrition to the risk of later-life metabolic disorders including T2D. In animal models of IUGR, changes in both DNA methylation and expression levels of key metabolic genes were repeatedly found which persisted until adulthood. The causal link between epigenetic disturbances during development and the risk for T2D was also confirmed in several human studies. In this review, the conceptual models and empirical data are summarized and discussed regarding the contribution of epigenetic mechanisms in developmental nutritional programming of T2D.

## 1. Introduction

Diabetes mellitus is one of the major causes of death across the globe. Type 2 diabetes (T2D), previously referred to as adult-onset or non-insulin-dependent diabetes, accounts for around 90% of all diabetes cases worldwide [1]. Presently, T2D being one of the most common noncommunicable disorders causes many serious health and social problems in both developing and developed societies. During the last decades, T2D has emerged as a global epidemic, with about 425,000 new cases estimated to occur annually [2]. Today, 415 million persons, i.e., about 9% of the worldwide adult population, have this disease, and that number is estimated to rise to 642 million persons throughout the next decade. Major risk factors contributing to the development of this pathological condition include genetic predisposition, stressful life conditions, unhealthy dietary behaviors, and low physical activity [3]. The pathophysiology of T2D is characterized by declining *β*-cell function, impaired glucose metabolism in the liver, and peripheral insulin resistance, a state in which insulin-responsive tissues exhibit reduced responsiveness to normal insulin concentrations [4, 5]. In order to compensate for insulin resistance to maintain normal glucose concentrations, *β*-cells produce more insulin but ultimately fail to do so and T2D may be diagnosed. The insulin resistance status is generally resulted from sedentary lifestyle and obesity occurring with increased age. Therefore, T2D is commonly regarded as an adult-onset disease typically occurring after the age of 40, although it is increasingly diagnosed now in more young patients [6].

Even though genetic factors definitely play a crucial role in driving T2D, the dramatic rise in incidence of this disease across countries apparently cannot be explained by genetic predisposing risk factors only but most likely depends on environmental and lifestyle conditions as well [3]. In recent years, consistent evidence is obtained that the risk for developing T2D and other metabolic disorders may be affected not only by adult-life environmental factors (primarily, those related to lifestyle) but also by early-life living conditions (for reviews, see Refs. [7–10]). A good example evidencing that nongenetic factors could contribute to the etiology and pathogenesis of this disease is research conducted in nuclear families of Pima Indians in which at least one of the siblings was born before and other(s) after the mother has been diagnosed with T2D [11]. In this study, those siblings conceived after the mother was diagnosed with diabetes had 3.7-fold higher risk to have T2D in comparison with siblings born before their mother became diabetic, even though their living conditions were highly similar during the rest of life. Another supportive evidence for importance of nongenetic factors in early-life etiology of T2D proceeds from the fact that monozygotic twins who are smaller at birth tend to have increased risk of developing T2D in adulthood [12] and also from the seasonality of birth in T2D patients [13]. In the last years, accumulating evidence indicates that epigenetic regulation of gene expression plays a critical role in linking early-life adverse conditions to the risk of later-life diseases [14]. There is an overwhelming body of evidence supporting the importance of epigenetic factors in the development of metabolic disorders including obesity, cardiometabolic disease, nonalcoholic fatty liver diseases, and also T2D [15, 16]. In this review, the conceptual models and empirical findings are summarized and discussed on the contribution of epigenetic mechanisms in the initiation and progression of T2D.

## 2. Conceptual Considerations

The causal link between early-life unfavorable conditions and adverse health outcomes in later life has been repeatedly confirmed in experimental studies and epidemiological observations. On the basis of these findings, the Developmental Origins of Health and Disease (DOHaD) concept was proposed postulating that both structure and physiology of the developing organism can be adapted to unfavorable growth conditions in such a way that predisposes to various pathological conditions in adulthood [17]. For instance, poor nutritional status during development may trigger structural and functional alterations in key organs, such as the liver, brain, muscle, pancreas, and adipose tissue, which can persist throughout life [18]. This view is currently often referred to as “predictive adaptive response” (PAR) hypothesis that describes the processes by which the developing organism draws from early-life experience to express a phenotype maximizing fitness basing on the expected future environmental conditions [19]. In particular, poor or unbalanced nutrient intake during intrauterine development can impair fetal growth resulting in intrauterine growth restriction (IUGR) and also cause fetal adipose tissue and pancreatic beta-cell dysfunction. As a result, the fetus may adapt to malnutrition by reducing capacity to produce insulin and by occurrence of insulin resistance. These processes can also result in “developmental programming” the appetite regulation and feeding behavior in adult life [8]. Such metabolic adaptations could provide short-term survival benefits in poor postnatal environments in consequence of elevated ability to store fat in the conditions of irregular accessibility of food resources. Realization of such individual adaptive strategy can, however, predispose to the development of T2D in conditions of abundant food supply in postnatal life. These considerations formed the basis for the “thrifty phenotype” hypothesis proposed by Hales and Barker in 1992 [20]. This concept followed from the thrifty genotype hypothesis proposed by Neel as early as in 1962 [21], which stated that the same genes that allowed to survive occasional famines anciently are being presently challenged by modern life conditions in which food is generally plentiful. Within the thrifty phenotype hypothesis, it is assumed that if a developing fetus is malnourished in consequence of suboptimal maternal nutrition, stress, placental dysfunction, or other unfavorable factors, this may result in adaptive response aimed at maximizing the metabolic efficiency in order to use and storage of nutrients. In developing these ideas, the thrifty epigenotype hypothesis was more recently proposed by Stöger, postulating that metabolism can develop into the healthy norm under normal dietary conditions; exposure to malnutrition in utero, however, results in compensatory epigenetic alterations in networks of energy and adipogenic metabolism genes which can, in turn, modify metabolism in such a way that resulting phenotype is better adapted for survival [22].

**Figure 1:**
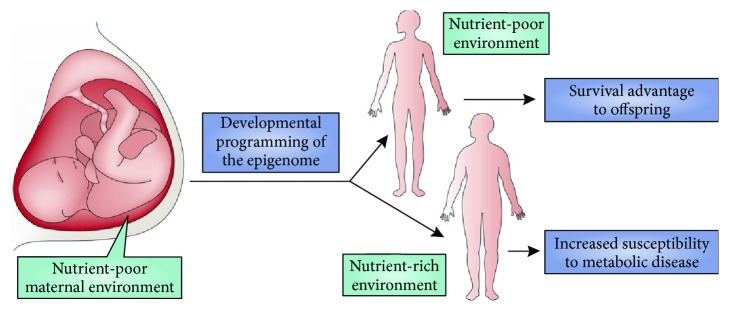
The thrifty phenotype hypothesis. Plasticity of the epigenome during development affords an opportunity for the developing organism to “preadapt” to the future adult environment, which provides a survival advantage. However, in settings in which the fetal environment does not match the adult environment—for example, fetal development in a nutrient-poor environment (such as maternal starvation) coupled with a nutrient-rich adult environment—the resulting “catch-up” growth and disconnection between fetal programming and the adult environment can predispose to adult metabolic disease, including obesity and type II diabetes. This figure and its legend are reproduced from Walker and Ho [30] with permission from Nature Publishing Group.

In IUGR conditions, the fetal adaptation to malnutrition may be realized by a number of mechanisms related to the glucose and energy metabolism, including elevated insulin sensitivity of peripheral tissues for glucose utilization, decreased insulin sensitivity for protein synthesis in muscles, impaired pancreatic development, and increased hepatic glucose production [23]. These processes provide apparent survival benefits for the IUGR fetuses by reducing the anabolic hormone production and demand for amino acids, promoting energy uptake and utilization, and increasing glucose production to maintain delivery of glucose to vital organs, primarily the brain [24]. Such adaptations result in asymmetrical growth of the fetus, with greatest restriction in muscles and subcutaneous tissues, less in bone tissue, and least in the brain [23]. In line with these theoretical considerations, significant reductions in fetal pancreatic tissue and in insulin-producing beta-cells have been found in rodent models [25, 26] and also in humans [27] that developed in severe IUGR conditions. On account of these processes, persisting adaptive changes can occur in the glucose-insulin metabolism. These changes can include reduced ability for insulin secretion and insulin resistance, and they may result in an improved capacity to store fat. In addition, restricted nutrient intake throughout in utero development may lead to long-term changes in appetite regulation and feeding behavior [28]. Such adaptive functional alterations are commonly accompanied by corresponding alterations in growth trajectory, i.e., low birth weight followed by rapid postnatal weight gain (“catch-up” growth) [29]. Collectively, these adaptations allow fetal tissues to maintain basic energy-dependent metabolic pathways at the expense of linear body growth in conditions of lowered food supply. However, if these modifications persist until adulthood or if they are more readily inducible in later life, they can support energy absorption beyond metabolic capacity in case if energy supply increases, thereby leading to the development of insulin resistance, obesity, and T2D in adult life ([22], see also Figure 1 for illustration).

In most early observational studies, birth weight was used as a proxy for IUGR. Initially, it has been suggested on the basis of findings obtained that low birth weight (LBW) is an important risk factor for T2D development. Proceeding from these assumptions, it has been assumed for a long time that there is a strong inverse linear relationship between birth weight and later risk of T2D [31]. The results of subsequent meta-analyses on the topic are, however, rather contradictory. In a recent meta-analysis, U-shaped relationship between birth weight and risk for T2D was revealed with high birth weight (HBW, >4,000 g) associated with an elevated risk of T2D to the same extent as LBW (<2,500 g) [32]. More recent meta-analysis has indicated that HBW is associated with a higher risk of nondiabetic obesity, but not T2D [33]. This ambiguity may be perhaps explained by the fact that association between LBW and risk for T2D in later life is mediated by catch-up growth (a greater-than-normal linear growth rate for chronological age following a period of growth inhibition) in early postnatal life of IUGR infants [34]. An important point is that catch-up growth results in a disproportionately increased rate of fat gain compared to lean tissue gain [35]. Such a preferential catch-up fat is driven by elaborate mechanisms of energy conservation operating via suppression of thermogenesis and leading to development of thrifty “catch-up fat” phenotype which is typically characterized by both leptin and insulin resistance. An epidemiological evidence for such a causal relationship was obtained, for example, in research by Eriksson and coauthors. In this research, LBW persons whose weight caught up in early childhood in the way that they had an average or above average weight from the age of seven years exhibited higher risk of developing hypertension and T2D and also higher coronary heart disease death rates in adulthood compared with their age-matched counterparts [36, 37].

## 3. Mechanistic Basis for Developmental Programming of T2D

**Figure 2:**
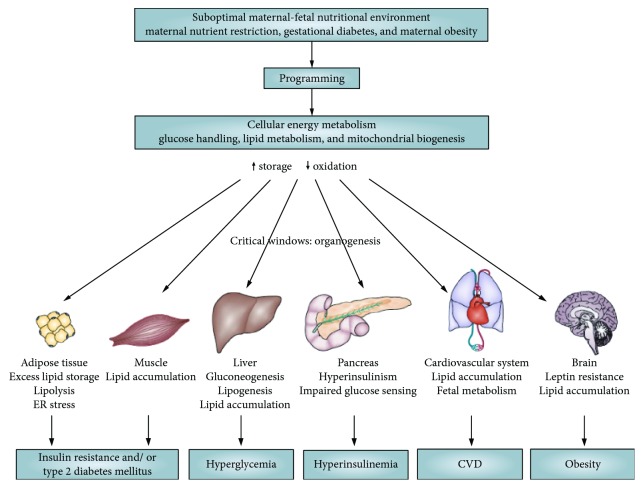
The contributory role of the maternal-fetal environment in the developmental programming of cellular energy metabolism in favor of lipid storage. This storage predisposes individuals to the metabolic syndrome. ER: endoplasmic reticulum. This figure and its legend are reproduced from Symonds et al. [42] with permission from Springer Nature.

Accumulating evidence suggests that long-term structural or/and functional changes can be induced in different organs of IUGR infants which permanently modulate their body functions [38, 39]. These alterations usually occur throughout the critical periods of early development, when processes of cell proliferation and differentiation reach their peaks and organs begin to form [40]. Retarded growth and restricted organ development are typically mediated by a reduction in cell number and an impaired balance of various cell types within tissues; such depletion in a set of the functional units within particular organs can subsequently impair their functional capability [41]. Main tissue and organ changes involved in the developmental programming of cellular energy metabolism in IUGR offspring are presented in Figure 2.

One good example for such changes is the development of the pancreas. The pancreas is an organ that is particularly sensitive to nutritional imbalance during intrauterine organogenesis. Inadequate nutrient intake throughout this stage can result in life-long structural and functional pancreatic tissue alterations. A substantial reduction in beta-cell mass and islet vascularization was revealed in various animal models, such as the rodent maternal calorie or protein restriction and also intrauterine artery ligation models [43]. Such persisting alterations in vital organs may definitely cause long-term modifications in various biochemical and/or hormonal pathways, thereby increasing the susceptibility to the development of particular pathological conditions in later life [44]. For example, in a rat model, prenatal maternal low-protein diet promoted cellular differentiation via upregulation of transcription factors. This resulted in stimulation of differentiation at the expense of proliferation in the neonatal pancreas and led to decreased beta-cell reserve, thereby likely contributing to a predisposition to T2D in later life [45]. The reduction in the islet cell mass and the relative proportion of beta-cells within the islets, as well as in the total pancreatic weight, were also observed in the IUGR rat offspring [46].

Precise molecular mechanisms underlying developmental programming of T2D are not thoroughly identified yet. Over the past decades, conclusive evidence has been provided for the central role of epigenetic modification of gene expression (heritable alteration in gene function without changes in the underlying DNA sequence) in these processes [13, 47, 48]. Whereas DNA is known to be relatively stable during the development of the organism, the epigenome (a totality of epigenetic marks across the entire genome) is changing dramatically throughout the fetal development to initiate differential patterns of gene expression among differentiating tissues. Main components of the “epigenetic code” that serves to fine-tuning of genetic circuit regulatory components comprise methylation of DNA and histone modifications which contribute to packing the DNA by forming nucleosomes.

In mammalian species including humans, DNA methylation is the most widely studied mechanism of epigenetic modification. This epigenetic mechanism consists of the addition of a methyl group at the 5^th^ position of the cytosine ring [49]. Methylation of CpG islands in gene promoters generally results in transcriptional silencing, although some transcription factors important for cell reprogramming throughout development have been recently identified which prefer to bind to CpG-methylated sequences [50]. Another key mechanism of epigenetic regulation is posttranslational modification of core histones, such as acetylation, methylation, phosphorylation, ubiquitination, and sumoylation of histone tails [51]. The “histone code” is formed by different combinations of histone modifications that mark the functional chromatin units, thereby recruiting coactivators/cosuppressors and transcription factors that regulate chromatin structure and gene activity [52–54]. The major enzymes catalyzing these changes are histone methyltransferases (HMTs), histone acetyltransferases (HATs), and histone deacetylases (HDACs) [55]. The processes of histone modification and DNA methylation are closely interrelated with each other. Histone modifications influence DNA methylation and vice versa, thereby collectively affecting chromatin accessibility to RNA polymerase and various transcription factors.

**Figure 3:**
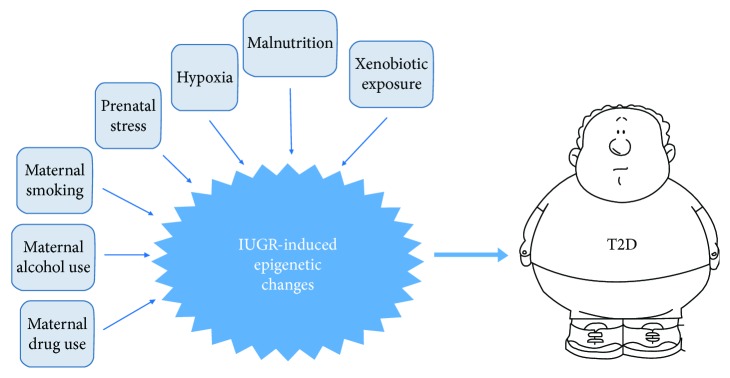
Schematic illustration of the association between adverse early-life exposures and the risk for developing T2D in adulthood.

An important point in the context of developmental programming is that epigenome is most plastic and sensitive to environmental stimuli during early development, particularly throughout the establishment of differentiation-dependent patterns of gene expression [56, 57]. Developmentally established epigenetic marks are stably maintained in different cell types throughout the life course. In mammals, including humans, the window of developmental epigenetic plasticity extends from preconception through weaning [57]. Such a nongenomic tuning of phenotype through developmental epigenetic plasticity has adaptive value since it attempts to match individual's responses to the environments predicted to be experienced [58–60]. However, when these responses are mismatched, it can result in an increased risk of disease. This underlies the “first 1000 days” concept prioritizing pregnancy and first two years of the child's life as a critical stage of human development [61]. In particular, early-life exposure to various adverse environmental factors may result in an increased risk for developing T2D in adult life. Among these factors, there are malnutrition, prenatal exposure to hypoxia, stress, and xenobiotics such as endocrine disruptors, as well as maternal smoking and consumption of alcohol and/or drugs during pregnancy (see Figure 3 for a schematic illustration). In this review, we will focus on the role of epigenetic mechanisms in mediating programming effects of prenatal malnutrition (both quantitative and qualitative) on subsequent risk for T2D. In subsequent sections, evidence from animal and human studies is provided for the role of epigenetic factors in developmental programming of T2D.

## 4. Research Evidence for Epigenetic Contribution to Prenatal Nutritional Programming of T2D

### 4.1. Evidence from Animal Models

Convincing evidence for the involvement of epigenetic factors in developmental origin of T2D comes from different animal species such as rodents, sheep, and nonhuman primates. In these studies, various models were used including maternal nutrient deficit or excess, uterine artery ligation, and exposure to toxic chemicals like phthalate or bisphenol A. Moreover, models of metabolic disturbances during pregnancy induced by maternal obesity and gestational diabetes mellitus were applied. These prenatal exposures caused substantial structural and functional alterations associated with abnormal glucose homeostasis in the pancreas, skeletal muscle, liver, and adipose tissue of the offspring. In these models, profound changes in expression of genes encoding key transcription factors, glucoregulatory enzymes, and nutrient receptors and transporters, playing a central role in the peripheral glucose uptake, development of pancreas and beta-cell function, and also insulin resistance were observed in IUGR offspring [15]. These epigenetic alterations may likely represent a potential mechanism by which suboptimal developmental conditions can result in an enhanced risk of developing T2D in offspring.

A causal link between developmental nutrition and T2D in adulthood was intensively studied using rodent models of 50% dietary restriction (DR) throughout gestation [62]. Rat offspring exposed to intrauterine DR had reduced beta-cell mass both at birth and throughout their early postnatal development [63–65]. Moreover, these rats were unable to adaptively increase beta-cell mass in adulthood in response to enhancing metabolic demands and consequent insulin resistance. As a consequence, they developed diabetic phenotypes characterized by failure of *β*-cells following impaired insulin secretion, insufficient expansion of beta-cell mass, glucose intolerance, and fasting hyperglycemia [66, 67]. These processes were found to be accompanied by profound epigenetic changes in key genes involved in *β*-cell development. For example, maternal DR caused significant reduction in expression of genes encoding key transcription factors contributing to embryonic beta-cell development such as the pancreatic and duodenal homeobox 1 (*Pdx-1*) gene in rats [68]. These epigenetic changes were associated with reduced beta-cell formation in postnatal life and inability to expand beta-cell mass in response to metabolic stresses. Lowered expression levels of *Pdx-1*, as well as other genes encoding transcription factors involved in regulating gluconeogenesis such as *FoxO1* and *MafA* genes, and also altered expression levels of miRNAs contributed to pancreatic development which were revealed in IUGR rat pancreas in the Zhang et al.'s study [69].

The maternal low-protein diet (LPD) model is another widely used experimental model in examining mechanisms underlying developmental programming. There are consistent similarities between findings from this model and those from studies of patients with T2D and other aspects of the metabolic syndrome [70]. The LPD model is based on ad libitum feeding to rodent dams, a diet containing 5-9% protein (casein) and a little under half the protein content but equivalent in energy of a standard control diet containing 18-20% protein [70, 71]. Maternal LPD resulted in decreased transcriptional activity of *Hnf4β*, a key transcription factor for beta-cell differentiation and glucose homeostasis, subsequently causing glucose intolerance in pancreatic islets of rat adult offspring [72]. Gestational LPD affected the expression of key metabolic genes such as *Igf2*, *GR*, *Nr3c1*, *Pparα*, and *Cyp2c34* [73–76], as well as genes involved in amino acid response pathway [77] in the liver of offspring rats. Maternal LPD during pregnancy also resulted in an increased expression of glucoregulatory genes like phosphoenolpyruvate carboxykinase (*PEPCK*) [78] and in a reduced expression of nutrient transporters including glucose transporter, *GLUT4* [79–81], thereby predisposing to insulin resistance in adult life. These changes in gene expression were found to be associated with persisting epigenetic changes consisting of histone code modifications such as elevated activity of histone deacetylases HDAC1 and HDAC4, increased binding of DNMT3a and DNMT3b, deacetylation of histone 3 lysine 14 (H3K14), increased recruitment of heterochromatin protein 1*α*, and demethylation of H3K9 (H3K9me2) in adult life [81]. In a mouse model, maternal LPD leads to a lower birth weight and also to impaired glucose tolerance and decreased insulin sensitivity at weaning [82]. These metabolic impairments were accompanied by profound epigenetic changes. More specifically, 253 differentially expressed genes mapped to 11 pathways have been identified in the livers of the offspring of LPD dams. Moreover, maternal LPD caused DNA demethylation in the promoter region of the leptin gene, thereby influencing feeding behavior and metabolic status in adulthood [83].

### 4.2. Evidence from Human Studies

Human data confirming the causal link between epigenetic disturbances during development and risk for metabolic disorders including T2D in adulthood are still scarce compared with findings from animal studies, primarily because of restricted access to appropriate human biological materials. It is accumulating evidence, however, that these mechanisms operate in human beings as well [14, 15, 84]. Such evidence is obtained, for example, in research of pancreas samples from deceased donors with T2D. In these studies, pervasive genome-wide epigenetic changes were observed in all levels of epigenetic regulation, including DNA methylation and histone modifications, and also in miRNA profiles [85–88]. However, since these changes may depend not only on early-life conditions but also on a variety of adult-life events, they are rarely discussed in the context of developmental origin of T2D. Most conclusive arguments in favor of developmental origin of T2D are derived from studies conducted with accessible perinatal tissues, such as the umbilical cord blood and placenta, with subsequent extrapolation of revealed epigenetic effects on the adult-life target tissues. However, the definitive conclusions about the developmental causality of these epigenetic alterations cannot be made from these studies. Indeed, since epigenetic alterations are to a large degree cell type- and tissue-specific and change in particular cell or tissue types can often not reflect a similar modification elsewhere, such an extrapolation may be fallacious [15]. Nevertheless, even with these limitations, such studies provide a valuable opportunity to obtain additional information on epigenetic factors potentially contributing to developmental programming of T2D. The epidemiological findings suggestive of the role of epigenetic factors in mediating the relationship between early-life experiences and risk for T2D in adulthood are reviewed and discussed in subsequent sections.

Since longitudinal designs are not possible in studying associations between early-life adverse exposures and later-life risk for T2D development in humans, the information about these associations comes mainly from observational studies conducted with a quasiexperimental design. Such studies (“natural experiments”) are defined as “naturally occurring circumstances in which subsets of the population have different levels of exposure to a supposed causal factor, in a situation resembling an actual experiment where human subjects would be randomly allocated to groups” [89]. The relationship of the T2D risk with unfavorable early-life events was well established in famine studies across different countries, including Holland [90, 91], Austria [92], Ukraine [93], and China [94–96]. In researching long-term health outcomes of prenatal exposure to Dutch famine (1944-45), DNA methylation changes potentially mediating these effects were determined. While no association was reported between the prenatal exposure to this famine and overall DNA methylation in adulthood [97], methylation levels of several genes in the adult offspring's whole blood samples were clearly associated with prenatal exposure to famine. Among them, genes known to be associated with development of metabolic and cardiovascular phenotypes, such as *IGF2* [98], and also *GNASAS*, *IL10*, *LEP*, *ABCA1*, *INSIGF*, and *MEG3* [99], were found to be differentially methylated between exposed individuals and nonexposed control persons six decades after the famine exposure. More recently, in a genome-scale analysis of differential DNA methylation in whole blood, it has been found that periconceptional exposure to famine resulted in differential methylation of genomic regions extended along pathways related to growth and metabolism [100]. Early gestation, but not mid or late gestation, was identified as a critical time period for inducing DNA methylation changes which can persist up to adulthood in whole blood of the perinatally exposed persons [101]. Remarkably, even though it has not been reported whether it was a correlation between DNA methylation and gene expression levels, the observed changes in DNA methylation were clearly associated with impaired metabolic homeostasis in adult subjects prenatally exposed to famine [102]. Similarly, in a historical cohort study performed in rural Bangladesh, offspring perinatally exposed to famine were found to be at higher risk of developing T2D and obesity in their adulthood compared to unexposed controls. Periconceptual famine-induced differences in DNA methylation were revealed at previously identified metastable epialleles sensitive to such exposure, including *VTRNA2-1*, *PAX8*, *PRDM-9*, near *ZFP57*, near *BOLA*, and *EXD3* [103].

## 5. Conclusions and Perspectives

Numerous experimental and epidemiological studies have provided consistent evidence linking unfavorable early-life conditions, such as developmental exposure to malnutrition or xenobiotics, with an increased risk for developing T2D and associated conditions in adulthood. Recently, data have been obtained suggesting that mechanisms involved in epigenetic regulation of gene expression can largely contribute to developmental etiology of T2D. Over recent years, epigenetic factors mediating these processes have been the subject of in-depth study, and several epigenetic mechanistic pathways potentially contributing to developmental metabolic programming have been identified. However, important outstanding issues have to be further addressed to better understanding cause-effect relationships underlying these processes. It is not clear so far to what extent developmentally induced epigenetic modifications can be translated to changes in gene expression. Indeed, changes on these levels often cooccur, but it is yet unknown whether this relationship is always causal. Moreover, it is still not fully established to what extent changes in gene expression can be translated into corresponding changes in protein content and activity and, accordingly, into alternative health/disease phenotypes. It is also still not clear how consistently reproducible are early-life-induced epigenetic modifications and whether they can persist until older ages when T2D usually manifests. There is some evidence that these modifications can persist life-long, thereby determining the risk of developing aging-related diseases like T2D [104, 105]. The evidence confirming the persistent character of these modifications is, however, still scarce. Therefore, further studies are required for better elucidating the molecular mechanism and signaling pathways underlying such long-term effects. One more methodological problem is that epigenetic profiles are highly tissue-specific [106]. Therefore, since epigenetic modifications originate both within and between different tissues, one important issue is applicability of data from peripheral blood or buccal swab samples to draw definitive conclusions. The study of tissues and organs which most significantly contribute to the pathogenesis of T2D would be of great interest. However, such tissues may in most cases be obtained from deceased donors only. Therefore, animal models that provide an opportunity of simultaneous characterization of epigenetic patterns in both peripheral and central tissues are highly useful in elucidating epigenetic pathways involved in developmental programming of T2D. The use of animal models, however, raises issues regarding the specificity of these pathways among mammalian species and also regarding similarities and distinctions between these pathways in different animal species and in man.

However, in spite of these unresolved issues, further investigation of epigenetic mechanisms contributing to developmental programming of T2D seems highly promising. Indeed, since epigenetic alterations, unlike genetic mutations, are potentially reversible [107], pharmacological correction of developmentally disrupted epigenetic patterns may provide a novel promising approach to prevention and treatment of T2D and associated disorders [108, 109]. Therefore, the implementation of new knowledge about the epigenetic pathways contributing to early etiology and pathogenesis of T2D would certainly be highly useful in clinical practice.
